# Mathematical modeling of metal recovery from E-waste using a dark-fermentation-leaching process

**DOI:** 10.1038/s41598-022-08106-2

**Published:** 2022-03-11

**Authors:** Fabiana Russo, Vincenzo Luongo, Maria Rosaria Mattei, Luigi Frunzo

**Affiliations:** Department of Mathematics and Applications “R. Caccioppoli”, University of Naples Federico II, Via Cintia 1, Monte S. Angelo, 80126 Naples, Italy

**Keywords:** Biological techniques, Biological models, Applied mathematics

## Abstract

In this work, an original mathematical model for metals leaching from electronic waste in a dark fermentation process is proposed. The kinetic model consists of a system of non-linear ordinary differential equations, accounting for the main biological, chemical, and physical processes occurring in the fermentation of soluble biodegradable substrates and in the dissolution process of metals. Ad-hoc experimental activities were carried out for model calibration purposes, and all experimental data were derived from specific lab-scale tests. The calibration was achieved by varying kinetic and stoichiometric parameters to match the simulation results to experimental data. Cumulative hydrogen production, glucose, organic acids, and leached metal concentrations were obtained from analytical procedures and used for the calibration. The results confirmed the high accuracy of the model in describing biohydrogen production, organic acids accumulation, and metals leaching during the biological degradation process. Thus, the mathematical model represents a useful and reliable tool for the design of strategies for valuable metals recovery from waste or mineral materials. Moreover, further numerical simulations were carried out to analyze the interactions between the fermentation and the leaching processes and to maximize the efficiency of metals recovery due to the fermentation by-products.

## Introduction

Over the last decades, the growing production and usage of electronic and electrical equipment both for commercial and domestic purposes resulted in a fast replacement of any type of electronic device^1–3^. Consequently, a huge amount of waste was generated at an alarming rate, when obsolete technologies are disposed and substituted^1,2^. This class of waste is known as electronic waste (E-waste). In recent years, different management strategies for E-waste are increasingly attracting the interest of the scientific community. This is due to the wide variety of materials contained in E-waste, which are potentially dangerous for the human health and environment. Indeed, E-waste is characterized by the presence of huge amounts of metals, such as copper (Cu), cobalt (Co), nickel (Ni), and lithium (Li)^4,5^. At the same time, they can contain valuable metals, such as gold (Au), and silver (Ag)^1^. Then, the definition of an adequate management strategy which allows to avoid pollutants release into the environment, and to recover and reuse valuable metals from E-waste is a crucial topic in both academic and industrial research. Nowadays, the most used methods for E-waste treatment and metals recovery are pyrometallurgical or hydrometallurgical processes. In pyrometallurgical processes, waste is usually burnt off, causing high energy consumption and emission of hazardous gases^6^. On the other hand, hydrometallurgical processes just use aqueous solutions during the E-waste treatment. For this reason, the latter are generally preferred at a large scale^7,8^. Indeed, hydrometallurgical processes have some attractive advantages, such as high recovery rates of metals, low consumption of energy, and minimal gas emission^3,7,9^. Among hydrometallurgical treatments, the most common strategy is the leaching process, which consists in the metals dissolution into the liquid phase. In this process both inorganic and organic acids can be used as leaching agents. Nevertheless, the leaching process catalyzed by inorganic acids has several environmental disadvantages, such as considerable emission of toxic gases, and water and soil contamination^3,6,9,10^. Indeed, residual compounds of the leaching process, including the liquid fraction, should be pretreated before their disposal^3,10–12^. Compared with inorganic acids, organic acids can be easily degraded and recycled, and their utilization as leaching agents does not cause secondary pollutants production^13^. For this reason, the liquid fraction is not considered potentially dangerous for the environment^3,6,7,14^ when organic acids are used for the leaching process.

Recently, the effective application of leaching processes using inorganic^15–17^ and organic^9,13,18^ has been proven in lab-scale. The latter evidence suggests that metals dissolution can be achieved using organic acids (OAs) produced by the dark fermentation (DF) process, resulting from the degradation of biodegradable substances with a contextual hydrogen production. Indeed, DF is a biological anaerobic process, which allows to the conversion of carbohydrates rich substrates into hydrogen ($H_2$), and other organic compounds, such as volatile fatty acids (VFAs). It represents a promising technology for waste valorization, as organic waste can be used as feeding substrate with consequent production of a renewable energy source. Usually, DF complexity depends on the specific microbial species involved in the bioprocess and on the biodegradability and composition characteristics of the substrate. On the other hand, it is a common practice referring to simple biodegradable compounds to compare hydrogen yields and effluent composition with experiments on raw waste biomasses. During the fermentation process, glucose is mainly converted into butyric and acetic acids. Such OAs are able to react with metals contained in the solid E-waste, and produce metallic compounds dissolved in the liquid effluent (Supplementary Fig. S1). The utilization of a VFAs-rich effluent as a leaching solution represents a suitable strategy to recover metals from both economical and environmental points of view. The residual VFAs can be processed with subsequent biological treatments for DF effluents. For instance, photofermentation (PF) allows to obtain an additional source of biohydrogen due to VFAs degradation by specific light dependent Purple Bacteria^19–21^, while anaerobic digestion (AD) leads to the conversion of organic acids in a methane-rich biogas^19^. Therefore, the integration of the leaching with the DF process represents a promising strategy for metal recovery as: (i) organic acids produced in the fermentative process avoid the utilization of chemicals; (ii) no external energy supply is required for metals recovery; (iii) PF or AD can be adopted for the leachate downstream treatment with the production of a renewable energy source (hydrogen or methane); (iv) the use of organic waste to feed the DF stage leads to biomass valorization in the biorefinery context; (v) any additional gaseous compounds can be trapped avoiding toxic emissions.

The integration of the DF with the leaching process still requires high research efforts, mainly due to the uncertainty and the limited knowledge on inhibition/stimulation dynamics generated by metals in DF reactors. In this framework, mathematical modeling represents a useful tool for investigating innovative and still poorly known processes^22^; it allows for testing a wide range of environmental conditions avoiding experimental tests, and for designing the correct management of any-scale applications. Moreover, it can be used to combine the DF and the leaching processes with the aim of optimizing metal recovery efficiency and hydrogen production, and minimizing energy consumption. Despite the great interest in this field, there is a lack of mathematical models taking into account contextually organic substrates degradation and metals dissolution during the DF-leaching process. Several models were employed for metals recovery by leaching process from waste and mineral materials. Such models are known as geochemical models, and are usually based on computer software interfaces, such as PHREEQC^23–25^, Visual Minteq^26^ and ORCHESTRA^27–29^. These tools are able to calculate the equilibrium composition of a diluted aqueous system, and they are generally applied to determine metal concentrations in lab-scale experiments or natural ecosystems.

In this work, a mathematical model able to describe the evolution of an integrated DF-leaching is proposed. The model is based on mass balance equations for soluble substrates, particulate and gaseous components, and metals in solid and liquid form. It consists of a system of non-linear ordinary differential equations (ODEs) where the state variables only depend on time. The model is able to account for biological, chemical, and physical processes taking place during the DF, and the related physico-chemical dynamics of metals due to leaching mechanisms in anaerobic reactors. Biomass growth and decay, substrates degradation, acid–base equilibrium, liquid-gas transfer, metals dissolution/precipitation, inhibition/limitation due to the process conditions were originally included in the presented work. The mathematical model was calibrated based on experimental data achieved with ad-hoc lab-scale tests. Cumulative hydrogen production, glucose degradation, OAs accumulation, and metals concentration trends were monitored, and used to calibrate the model. The bioreators were carried out in batch conditions by using a synthetic solution of glucose, an anaerobic digestate, and spent button batteries, to provide a feeding organic substrate, a microbial inoculum, and a suitable metals source, respectively. Numerical simulations demonstrated the applicability of the model for an accurate prediction of the DF-leaching process. The calibrated model can be applied as an optimizing tool supporting lab-scale or higher scale applications. Four numerical studies were also presented aimed at optimizing the recovery of valuable metals from wastes or minerals, and to demonstrate the applicability of the model in the leaching process management. In particular, the numerical studies investigate three fundamental aspects: how metals inhibition affects the DF process; how *F*/*M* ratio affects the DF process and leaching efficiency; and how metal concentration affects the efficiency of the leaching process.

## Biochemical framework: dark fermentation and leaching processes

DF is a promising technology for biohydrogen production, due to its high production rate and hydrolytic effect on organic waste. In DF process, carbohydrates rich substrates are anaerobically converted by hydrogen-producing microorganisms to hydrogen and organic acids. DF is a complex biotechnology as many factors, such as bioreactor configuration, operating conditions, substrate to inoculum ratio and composition, inoculum pretreatment method, temperature, and pH, are able to influence metabolic pathways affecting the production rate and the effluent composition^19,30^. Organic substrate characteristics, e.g. carbohydrates content, bioavailability, and biodegradation rate, play an important role in the biohydrogen generation^19^. Glucose and sucrose are the most common substrates used for lab-scale DF experiments^19,21,30–32^. To provide a suitable mixed culture DF inoculum, cow dung, anaerobic sludge, municipal solid waste, and compost, are usually adopted as a source of microorganisms^19,21,30^. To enhance biohydrogen production and to inhibit hydrogen consumers (*methanogens*) activity, an adequate pretreatment strategy, such as thermal/chemical inoculum treatment, is required^30,32,33^. Temperature and pH are crucial parameters for fermentative processes^30^. Increasing temperature usually leads to microorganism selection and enhancement of hydrogen production rates, while a neutral pH is usually recommended for DF processes devoted to $H_{2}$ generation. Indeed, an acidic environment may inhibit the metabolic activity of hydrogen-producers microorganisms both in mesophilic (35 °C) or thermofilic (55 °C) conditions^19^. However, the most common temperature used in DF applications is 35 °C, as this condition positively affects the hydrogen production and limits the management costs due to energy supplementation to bioreactors^19^.

In DF processes, glucose $C_6H_{12}O_6$ is mainly converted to butyric acid $CH_3CH_2CH_2COOH$, acetic acid $CH_3COOH$, hydrogen $H_2$, and carbon dioxide $CO_2$^19,31,34^ during *acidogenesis*^35^. It can be assumed that *acidogenesis* and $H_2$ generation are contextually performed by sugar fermenters, whose metabolism is described by biochemical reactions:

1 $$\begin{aligned}&C_6H_{12}O_6+2H_2O \rightarrow 2CH_3COOH+2CO_2+4H_2, \end{aligned}$$

2 $$\begin{aligned}&C_6H_{12}O_6 \rightarrow CH_3CH_2CH_2COOH+2CO_2+2H_2. \end{aligned}$$

In glucose fermentation, enzymatic conversion processes involving composite particulate materials, such as *disintegration* and *hydrolysis*, can be neglected, as the organic substrate is already fed in soluble form. In addition, the *acetogenesis* and *methanogenesis* processes, usually occurring during the anaerobic digestion, are inhibited by inoculum pretreatment^19,30,33^. An additional contribution to *methanogenesis* inhibition is provided by the substrate, or food (F), to inoculum, or microorganisms (M), ratio. An high *F*/*M* favours organic acids production and accumulation in DF reactors, with consequent pH level reduction and inhibition of methane generation.

On the other hand, the leaching process is a hydrometallurgical treatment consisting of dissolution reactions. Solid metals contained in various wastes are converted to the liquid form due to the interaction with a leaching agent^6^. To this aim, inorganic acids, such as $H_2SO_4$, *HCl*, and $HNO_3$^6,7,36^, are commonly used, as they contextually allow for the E-waste treatment and metal recovery. Despite the high efficiency rate of inorganic acids in leaching processes, further treatments are required for the resulting acidic effluent. Moreover, hazardous gases are typically produced during the dissolution phase and a notable amount of inorganic acids, with significant market value, is required. To overcome these disadvantages, the use of organic acids as leaching agents for metal recovery was recently introduced. These biodegradable compounds can be obtained by organic waste fermentation, and are characterized by adequate acidity for the metal dissolution. Citric acid, oxalic acid, and acetic acid are the most common organic acids tested for the leaching process^3,11^. As reported in Golmohammadzadeh et al. (2018)^3^, acetic acid allows for the dissolution of a generic metal with the leaching reaction:

3 $$\begin{aligned} n[C_2H_3O^-_3]_{(liq)}+M^{n+}_{(s)} \rightarrow M[C_2H_3O_2]_{n_{(liq)}} \end{aligned}$$

where *n* represents the oxidation states of the metal. Once the dissolution is complete, the metals can be recovered from the leachate by different chemical methods, such as precipitation, solvent extraction, and electrolytic deposition^6,9,37^. However, precipitation represents the most common method adopted for the final metal recovery^6,7^.

## Methods

### Mathematical model

In this section, the complete mathematical model describing the integrated DF-leaching process for metals recovery is presented. The dissolution process is described by chemical reactions involving the organic acids produced in the DF process and the metals supplemented to the system. The reactor is modeled as a continuous stirred tank reactor (CSTR) with a constant total volume *V*. As shown in Supplementary Fig. S2, the reactor is constituted by the liquid and the gaseous phases. The mathematical model is able to account for all liquid-gas interactions occurring during the anaerobic fermentation, i.e. *acidogenesis*, $H_2$ production, biomass growth and decay, acid–base reactions, physical interactions, and leaching reactions. The proposed model is described by a system of nonlinear differential equations for soluble, particulate and gaseous components. These components, expressed in terms of concentration, are divided in: $n_1$ soluble substrates $S_i(t)$ involved in the biochemical processes; $n_2-n_1$ particulate materials $X_i(t)$, representing the microbial groups operating the biochemical conversion; $n_1$ gas components $S_{gas,i}(t)$ involved in the liquid-gas equilibrium; $n_3$ metals in solid $M_{i}(t)$ and liquid $M_{liq,i}(t)$ dominated by the leaching process. Such concentrations vary over time due to biological and chemical processes and operating parameters of the reactor. The differential equations governing soluble, particulate, gaseous components, and metals are described by Eqs. (4)–(8):

4 $$\begin{aligned}&\frac{dS_i}{dt} = \frac{Q^{in}S^{in}_i(t)}{V_{liq}}-\frac{Q^{out}S_i(t)}{V_{liq}}+\sum _{j=1}^{m_1}\alpha _{i,j}\rho _j(t,\mathbf{S },\mathbf{X },\mathbf{M })+\sum _{j=1}^{m_2}\beta _{i,j}\rho _{A,j}(t,\mathbf{S })+\nonumber \\&\quad +\sum _{j=1}^{m_3}\gamma _{i,j}\rho _{M,j}(t,\mathbf{S },\mathbf{M })-\rho _{T,i}(t,\mathbf{S }, \mathbf{S_{gas}}), \ i=1,\ldots ,n_1, \ t>0, \end{aligned}$$

5 $$\begin{aligned}&\frac{dX_i}{dt} = \frac{Q^{in}X^{in}_i(t)}{V_{liq}}-\frac{Q^{out}X_i(t)}{V_{liq}}+\sum _{j=1}^{m_1}\alpha _{i,j}\rho _j(t,\mathbf{S },\mathbf{X },\mathbf{M }), \ i=n_1+1,\ldots ,n_2, \ t>0, \end{aligned}$$

6 $$\begin{aligned}&\frac{dS_{gas,i}}{dt} = -\frac{Q_{gas}S_{gas,i}}{V_{gas}}+\frac{V_{liq}}{V_{gas}} \rho _{T,i}(t,\mathbf{S} ,\mathbf{S} _{\mathbf{gas}} ), \ i=1,\ldots ,n_1, \ t>0, \end{aligned}$$

7 $$\begin{aligned}&\frac{dM_{i}}{dt} = \frac{Q^{in}M^{in}_i(t)}{V_{liq}}-\frac{Q^{out}M_i(t)}{V_{liq}}+\sum _{j=1}^{m_3}\delta _{i,j}\rho _{M,j}(t,\mathbf{S },\mathbf{M }), \ i=1,\ldots ,n_3, \ t>0, \end{aligned}$$

8 $$\begin{aligned}&\frac{dM_{liq,i}}{dt} = \frac{Q^{in}M^{in}_{liq,i}(t)}{V_{liq}}-\frac{Q^{out}M_{liq,i}(t)}{V_{liq}}+\sum _{j=1}^{m_3}{\bar{\delta }}_{i,j}\rho _{M,j}(t,\mathbf{S },\mathbf{M }), \ i=1,\ldots ,n_3, \ t>0, \end{aligned}$$

where $m_1$, $m_2$, and $m_3$ denote the number of biochemical, acid–base and physico-chemical processes accounted in the mathematical model; $\rho _j(t,\mathbf{S },\mathbf{X },\mathbf{M })$ represents the kinetic rate equation for the *j*th biochemical process, respectively; $\rho _{A,j}(t,\mathbf{S})$ represents the kinetic rate equation for the *j*th acid–base reaction; $\rho _{M,j}(t,\mathbf{S },\mathbf{M })$ represents the reaction rate equation for the *j*th physico-chemical process; $\rho _{T,i}(t,\mathbf{S,S_{gas}})$ represents the rate equation for the liquid-gas transfer process of the *i*th component; $\alpha _{i,j}$ is the rate coefficient of the *i*th component referred to the *j*th biochemical process; $\beta _{i,j}$ is the rate coefficient of the *i*th component referred to the *j*th acid–base process; $\gamma _{i,j}$, $\delta _{i,j}$ and ${\bar{\delta }}_{i,j}$ are the rate coefficients of the *i*th component referred to the *j*th physico-chemical process; $\mathbf{S }=(S_1,\ldots ,S_{n_1})$, $\mathbf{X }=(X_{n_1+1},\ldots ,X_{n_2})$, $\mathbf{S }_{\mathbf{gas}} =(S_{gas,1},\ldots ,S_{gas,n_1})$, $\mathbf{M }=(M_{1},\ldots ,M_{n_3})$, and $\mathbf{M }_{\mathbf{liq}} =(M_{liq,1},\ldots ,M_{liq,n_3})$. Regarding the operating parameters, $Q^{in}$ and $Q^{out}$ are the inlet and outlet wastewater flow of the biological reactor; $Q_{gas}$ is the total gas flow; $S_i^{in}$, $X_i^{in}$, $M_i^{in}$ and $M_{liq,i}^{in}$ represent the influent concentrations of the *i*th solute, particulate component and metal in solid and liquid form, respectively; $V_{liq}$ and $V_{gas}$ are the liquid volume and the gas volume of the biological reactor and their sum gives the constant total volume *V*. Such mass balance equations represent a system of nonlinear ordinary differential equations, where the state variables depend on time, and the non-linearity is due to the reaction terms. The initial condition required to solve the system is reported in Eqs. (9)–(13),

9 $$\begin{aligned}&S_{i}(0)=S_{i}^{0}, \quad i=1,\ldots ,n_1, \end{aligned}$$

10 $$\begin{aligned}&X_{i}(0)=X_{i}^{0}, \quad i=n_1+1,\ldots ,n_2, \end{aligned}$$

11 $$\begin{aligned}&S_{gas,i}(0)=S_{gas,i}^{0}, \quad i=1,\ldots ,n_1, \end{aligned}$$

12 $$\begin{aligned}&M_{i}(0)=M_{i}^{0}, \quad i=1,\ldots ,n_3, \end{aligned}$$

13 $$\begin{aligned}&M_{liq,i}(0)=M_{liq,i}^{0}, \quad i=1,\ldots ,n_3, \end{aligned}$$

where $S_{i}^{0}$, $X_{i}^{0}$, $S_{gas,i}^{0}$, $M_{i}^{0}$ and $M_{liq,i}^{0}$ are the initial concentrations of the *i*th soluble substrate, particulate component, gas component, and metal in solid and liquid form, respectively.

### Model application

The mathematical model was applied to simulate metal recovery from spent button lithium-ion batteries (LIBs) during glucose fermentation. Ad-hoc experimental activities were set-up in batch conditions to achieve the required data for model calibration. Biochemical and physico-chemical processes occurring in DF experiments (metabolic pathways, acid–base reactions, liquid-gas equilibrium) as well as dissolution reaction kinetics of the leaching process were monitored during the tests. Based on experimental observations (Supplementary Information), different variables, in terms of concentrations, were accounted in the model: 10 soluble substrates (glucose $S_{su}$, butyric acid $S_{bu}$, acetic acid $S_{ac}$, hydrogen $S_{H_2}$, inorganic carbon $S_{IC}$, inorganic nitrogen $S_{IN}$, butyrate $S_{bu^-}$, acetate $S_{ac^-}$, bicarbonate $S_{HCO_3^-}$, ammonia $S_{NH_3}$); 1 particulate component (sugar fermenters $X_{su}$); 2 gas components (hydrogen gas $S_{gas,H_2}$, carbon dioxide $S_{gas,CO_2}$); manganese in solid form (*M*); manganese in liquid form ($M_{liq}$). Due to the batch condition adopted for lab-scale tests, the inlet and outlet wastewater flow were assumed to be equal to 0 ($Q^{in}=Q^{out}=0$), and Eqs. (4)–(8) were specified as:

14 $$\begin{aligned}&\frac{dS_{su}}{dt} = -\rho _1, \end{aligned}$$

15 $$\begin{aligned}&\frac{dS_{bu}}{dt} = (1-Y_{su})f_{bu,su}\rho _1-n\rho _{M,1}, \end{aligned}$$

16 $$\begin{aligned}&\frac{dS_{ac}}{dt} = (1-Y_{su})f_{ac,su}\rho _1, \end{aligned}$$

17 $$\begin{aligned}&\frac{dS_{H_2}}{dt} = (1-Y_{su})f_{H_2,su}\rho _1 - \rho _{T,H_2}, \end{aligned}$$

18 $$\begin{aligned}&\frac{dS_{IC}}{dt} = C_{su}\rho _1-(1-Y_{su})(f_{bu,su}C_{bu}+f_{pro,su}C_{pro}+f_{ac,su}C_{ac})\rho_1-Y_{su}C_{biom}\rho_1\nonumber \\&\quad +C_{biom}\rho_2 - \rho _{T,IC}, \end{aligned}$$

19 $$\begin{aligned}&\frac{dS_{IN}}{dt} = -Y_{su}N_{biom}\rho _1+N_{biom}\rho _2, \end{aligned}$$

20 $$\begin{aligned}&\frac{dS_{bu^-}}{dt} = -\rho _{A,bu^-}, \end{aligned}$$

21 $$\begin{aligned}&\frac{dS_{ac^-}}{dt} = -\rho _{A,ac^-}, \end{aligned}$$

22 $$\begin{aligned}&\frac{dS_{HCO_3^-}}{dt} = -\rho _{A,HCO_3^-}, \end{aligned}$$

23 $$\begin{aligned}&\frac{dS_{NH_3}}{dt} = -\rho _{A,NH_3}, \end{aligned}$$

24 $$\begin{aligned}&\frac{dX_{su}}{dt} = Y_{su}\rho _1-\rho _2, \end{aligned}$$

25 $$\begin{aligned}&\frac{dS_{gas,H_2}}{dt} = -\frac{Q_{gas}S_{gas,H_2}}{V_{gas}}+\frac{V_{liq}}{V_{gas}}\rho _{T,H_2}, \end{aligned}$$

26 $$\begin{aligned}&\frac{dS_{gas,CO_2}}{dt} = -\frac{Q_{gas}S_{gas,CO_2}}{V_{gas}}+\frac{V_{liq}}{V_{gas}}\rho _{T,IC}, \end{aligned}$$

27 $$\begin{aligned}&\frac{dM}{dt} = -\rho _{M,1}, \end{aligned}$$

28 $$\begin{aligned}&\frac{dM_{liq}}{dt} = \rho _{M,1}-\rho _{M,2}, \end{aligned}$$

where $Y_{su}$ is the yield of sugar fermenters; $f_{bu,su}$, $f_{ac,su}$, and $f_{H_2,su}$ represent the fractions of butyrate, acetate, and hydrogen generated from sugar fermentation, respectively; $C_{su}$, $C_{bu}$, and $C_{ac}$ are the carbon content of sugar, butyrate, and acetate, respectively; $C_{biom}$, and $N_{biom}$ are the carbon and nitrogen content of the biomass; and *n* represents the oxidation states of the metal (Eq. 3). The characterization of the spent button LIBs was carried out following the procedure proposed by Russo et al. (2022)^38^ (Supplementary Information). The internal part of batteries was mainly composed by: Manganese ($45.0\%$), Lithium ($9.45\%$), Silicon ($0.18\%$), Iron ($0.11\%$), Sodium ($0.13\%$), Calcium ($0.05\%$), Magnesium ($0.04\%$), Potassium ($0.03\%$), Nickel ($0.01\%$), Aluminium ($0.01\%$) and Chromium ($0.01\%$). The complete metals composition of the E-waste is reported in Supplementary Table S1. Some of these metals are potentially dangerous for human health and environment^39^. Specifically, among all the metals contained in LIBs, the experimental campaign focused on manganese leaching as it was one of the most abundant metal in the specific E-waste. For this reason, only the dissolution process of manganese was considered in the mathematical model, assuming that other metals contained in the waste did not take part in the dissolution process, and the butyric acid $S_{bu}$ consumption was related to the leaching process exclusively of manganese *M*. In particular, the most common oxidation state of manganese is $+2$ ($n=2$). Furthermore, according to the experimental data the concentration of the metal in the liquid form increased at the beginning of the leaching process, while a subsequent reduction was observed in the last experimental days. Based on this evidence, both the manganese dissolution ($\rho _{M,1}$) and subsequent reduction phase ($\rho _{M,2}$) were modeled.

#### Biochemical reaction rates

The DF process is performed by a single microbial group defined as sugar fermenters $X_{su}$. The growth process leads to the consumption and/or production of one or more soluble substrates, and a negative term was considered to account for microorganisms decay during the process. In particular, $X_{su}$ operate the glucose $S_{su}$ degradation and the contextual production of VFAs, such as butyric $S_{bu}$ and acetic acid $S_{ac}$, and hydrogen $S_{H_2}$. The kinetic rate equation related to the *acidogenesis* process $\rho _1$ in Eqs. (14)–(19) and Eq. (24) was considered as a Monod-type kinetic:

29 $$\begin{aligned} \rho _1= k_{m,su}\frac{S_{su}}{K_{su}+S_{su}} X_{su} I, \end{aligned}$$

while, the decay rate $\rho _2$ in Eqs. (18), Eq. (19), and Eq. (24) was modeled as a first order kinetic:

30 $$\begin{aligned} \rho _{2}= k_{dec,X_{su}}X_{su}, \end{aligned}$$

where $k_{m,su}$ is the Monod maximum specific uptake rate, which is achieved by dividing $\mu _{max,su}$ by $Y_{su}$, $\mu _{max,su}$ is the Monod maximum specific growth rate of sugar fermenters, $K_{su}$ is the affinity constant, $k_{dec,X_{su}}$ is the first order decay rate of sugar fermenters, and *I* represents an inhibition function depending on pH value, inorganic nitrogen limitation, and metal concentration within the bioreactor. The inhibition function is detailed in the following.

#### Leaching reaction rates

In both experimental sets, manganese *M* dissolution, induced by organic acids generation, was followed by a reduction of the liquid metal concentration due to precipitation/complexation phenomena. The reaction rate equation related to the dissolution process $\rho _{M,1}$ (Eqs. 15, 27 and 28) and the subsequent reduction of the metal concentration in soluble form $\rho _{M,2}$ (Eq. 28) were modeled by equations:

31 $$\begin{aligned}&\rho _{M,1}= k_{d} M^a S_{bu}^b. \end{aligned}$$

32 $$\begin{aligned}&\rho _{M,2}= k_{r} M_{liq}^c, \end{aligned}$$

where *a*, *b*, and *c* are the reaction order parameters, and $k_{d}$, and $k_{r}$ represent the dissolution and reduction constants of the leaching process, respectively.

The dissolution process (Eq. 31) was modeled as a first order kinetic referred to the manganese concentration in solid form *M* (*a* equal to 1), and a second order kinetic referred to the butyric acid $S_{bu}$ (*b* equal to 2). This assumption was supported by experimental evidences^13,17^ demonstrating that the metals leaching is strongly affected by the acid concentration in the case of both inorganic and organic acids. According to a previous study^37^, the subsequent decrease of soluble metal in the anaerobic environment was ascribed to adsorption or precipitation phenomena (Eq. 32). Due to the complexity of the reaction environment of experiments, from the available data it was not possible to distinguish the adsorption or the precipitation contributions during the dark fermentation-leaching process. For this reason, the reduction of metal in solution was simply modeled as a first order kinetic referred to the manganese concentration in solution (assuming the reaction order parameter *c* equal to 1). Indeed, other models considered the same approximation to reproduce metals precipitation and metals adsorption phenomena^40–42^.

#### Acid–base process rates

As mentioned above, pH and temperature play an important role in the evolution of the biochemical pathways involved in the DF process. To achieve high substrate degradation efficiency and $H_2$ yield, experimental findings showed that the pH level may vary from 4.5 to 7, and mesophilic temperatures (about 35 °C) are adequate for mixed culture fermentation^19^. During DF, high concentration of VFAs are produced, leading to the pH decrease in the reaction environment. Such acidification may lead to a partial or complete inhibition of microbial consortia, and may directly affect the $H_2$ generation rate. The acid–base equilibrium equations play an important role for pH calculation. In aqueous solution, any organic or inorganic compound leads to the production of acid–base pairs (e.g. proton $H^{+}$ and conjugate base) depending on the specific pH level. In order to simulate pH variation over time, a charge balance equation, accounting for all the dissolved ionic species, was considered in the mathematical model and was expressed as:

33 $$\begin{aligned} \sum _{i} \mathbf{S_{i}^{+}}-\sum _{i} \mathbf{S_{i}^{-}}=0, \end{aligned}$$

where $\mathbf{S_{i}^{+}}$ and $\mathbf{S_{i}^{-}}$ represent the cationic and anionic equivalent concentration of the *i*th component. The $H^+$ concentration $S_{H^+}$ was obtained solving Eq. (33), which takes the form:

34 $$\begin{aligned} S_{H^+}+S_{NH_4^+}-S_{HCO_3^-}-\frac{S_{bu^-}}{160}-\frac{S_{ac^-}}{64}-S_{OH^-}=0 \end{aligned}$$

where $S_{NH_4^+}$ is the $NH_4^+$ concentration given by the difference of the inorganic nitrogen $S_{IN}$ and the ammonia $S_{NH_3}$ concentrations in the system; $S_{IN}$, $S_{bu^-}$, $S_{ac^-}$, $S_{HCO_3^-}$, and $S_{NH_3}$ were obtained solving Eqs. (19)–(23). The kinetic rates defined for each acid–base equilibrium in Eqs. (20)–(23) are listed below:

35 $$\begin{aligned}&\rho _{A,{bu^-}}=K_{A/B,bu}(S_{bu^-}(S_{H^+}K_{a,bu})-K_{a,bu}S_{bu}), \end{aligned}$$

36 $$\begin{aligned}&\rho _{A,{ac^-}}=K_{A/B,ac}(S_{ac^-}(S_{H^+}K_{a,ac})-K_{a,ac}S_{ac}), \end{aligned}$$

37 $$\begin{aligned}&\rho _{A,{HCO_3^-}}=K_{A/B,CO_2}(S_{HCO_3^-}(S_{H^+}K_{a,CO_2})-K_{a,CO_2}S_{IC}), \end{aligned}$$

38 $$\begin{aligned}&\rho _{A,{NH_3}}=K_{A/B,IN}(S_{NH_3}(S_{H^+}K_{a,IN})-K_{a,IN}S_{IN}). \end{aligned}$$

where $K_{A/B,i}$ and $K_{a,i}$ are the acid–base kinetic parameter and the acid–base equilibrium coefficient for the *i*th component.

#### Physico-chemical processes

Liquid-gas transfer equations were used to describe hydrogen $S_{H_2}$ and inorganic carbon $S_{IC}$ evolution in the liquid and the gas phase of bioreactors. When the liquid phase is relatively dilute, Henry’s law can be used to describe the liquid-gas equilibrium. The liquid-gas transfer kinetic rates in Eq. (17), Eq. (18), Eq. (25), and Eq. (26) were expressed as:

39 $$\begin{aligned}&\rho _{T,H_2}=kLa(S_{H_2}-16K_{H,H_2}p_{gas,H_2}), \end{aligned}$$

40 $$\begin{aligned}&\rho _{T,IC}=kLa(S_{CO_2}-K_{H,CO_2}p_{gas,CO_2}), \end{aligned}$$

where *kLa* is the gas-liquid transfer coefficient, $K_{H,i}$ is the Henry’s law coefficient of the *i*th component, $p_{gas,i}$ is the steady-state gas phase partial pressure of the *i*th component, and $S_{CO_2}$ is the $CO_2$ concentration given by the difference of $S_{IC}$ and $S_{HCO^-_3}$. The computation of $p_{gas,i}$ is required to compute the mass transfer kinetic rates, and it is given by:

41 $$\begin{aligned} p_{gas}=p_{gas,H_2}+p_{gas,CO_2}+p_{gas,H_2O}, \end{aligned}$$

where:

42 $$\begin{aligned}&p_{gas,H_2}=S_{gas,H_2}\frac{RT}{16}, \end{aligned}$$

43 $$\begin{aligned}&p_{gas,CO_2}=S_{gas,CO_2}RT, \end{aligned}$$

44 $$\begin{aligned}&p_{gas,H_2O}=0.0313 \ exp\Big (5290\Big (\frac{1}{298}-\frac{1}{T}\Big )\Big ), \end{aligned}$$

while, the gas flow $Q_{gas}$ necessary to solve Eq. (25) and Eq. (26) was set equal to the total gas transfer:

45 $$\begin{aligned} Q_{gas}=\frac{RT}{p_{gas}-p_{gas,H_2O}}V_{liq}\Big (\frac{\rho _{T,H_2}}{16}+\rho _{T,CO_2}\Big ), \end{aligned}$$

where *R* and *T* are the gas law constant and temperature, respectively.

#### Inhibition functions

Several inhibition mechanisms were considered in the mathematical model to account for the influence of: (i) the pH level on the process evolution; (ii) the inorganic nitrogen concentration; (iii) the presence of metals. Then, the inhibition function *I* in Eq. (29) was expressed as follows:

46 $$\begin{aligned} I=I_{pH}I_{IN,lim}I_{L}. \end{aligned}$$

The inhibition term $I_{pH}$ describes that the pH level of the reaction environment directly affects metabolic activities of anaerobic bacteria. In accordance with previous studies^35^, the optimal range of pH values are between 5.5 and 7. To account of upper and lower inhibition pH level, the pH inhibition term $I_{pH}$ was implemented with the following empirical equation^35,43^:

47 $$\begin{aligned} I_{pH} = \frac{1+2\times 10^{0.5(pH_{LL}-pH_{UL})}}{1+10^{(pH-pH_{UL})}+10^{(pH_{LL}-pH)}}, \end{aligned}$$

where $pH_{UL}$ and $pH_{LL}$ are upper and lower limits. These represent the pH values which lead to a maximum growth rate reduction of $50\%$.

The inorganic nitrogen limitation term $I_{IN,lim}$ was included to describe the decrease of the maximum growth rate due to a reduced nitrogen $S_{IN}$ availability in bioreactors^35^. The limiting term $I_{IN,lim}$ was described as:

48 $$\begin{aligned} I_{IN,lim}=\frac{1}{1+\frac{K_{IN}}{S_{IN}}}, \end{aligned}$$

where $K_{S,IN}$ represents the $S_{IN}$ affinity constant for $X_{su}$.

Finally, the metals inhibition term $I_{L}$ was included to describe the condition in which high concentration of metals negatively affects the metabolic activity^44,45^. Metals, such as cadmium (Cd), chromium (Cr), zinc (Zn), copper (Cu), nickel (Ni), and manganese (Mn), may be present in E-waste and inhibit or upset the fermentative process due to the increase of the toxicity level of the environment. The presence of high concentrations of metals is able to reduce the hydrogen production rate by 50%^19^. The metals inhibition term $I_{L}$ was modeled with a non-competitive inhibition function^35^:

49 $$\begin{aligned} I_{L}=\frac{1}{1+\frac{M}{K_{L}}}, \end{aligned}$$

where $K_{L}$ is the inhibition constant related to the leaching process. The inhibition term $I_{L}$ assumes a value lower than 1 since the metal concentration *M* is greater than 0. In particular, the higher is the metal concentration, the stronger is the inhibition effect. On the contrary, if *M* is equal to 0, the inhibition term $I_{L}$ assumes a constant value equal to 1.

## Model calibration

Due to the scarcity of literature data related to the leaching process conducted with DF produced OAs, ad-hoc experimental activities were carried out for model calibration purposes. To investigate different interactions occurring between the biological and the chemical process investigated, it was necessary to set up 2 different initial conditions (IC) for bioreactors as reported in Supplementary Information. The E-waste was added at the beginning of the DF experiments (*IC*1) and when it was possible to consider that the DF process was completely developed (*IC*2). The aim of this strategy was to build-up a complete data set to calibrate all the different parameters related to metals inhibition, biological process evolution and leaching process. Indeed, in the *IC*1 the inhibition effects related to the presence of metals delayed the DF evolution, increasing the initial toxicity level in the bioreactors. On the contrary, in the *IC*2 no inhibition phenomena occurred at the beginning of the biological process. However, the metal dissolution was observed in both cases, but with different evolution trends. In addition, to allow the model for reproducing an impulsive addition of the E-waste at a specific time, Eq. (27) was replaced by the following impulsive ordinary differential equation (IDE):

50 $$\begin{aligned}&\frac{dM}{dt} = -\rho _{M,1}(t,\mathbf{S },\mathbf{M }), \ t \ne t_{W},\ t>0, \end{aligned}$$

51 $$\begin{aligned}&\Delta M(t_{W})=M(t_{W})=M(t^+_{W})-M(t^-_{W}), \end{aligned}$$

where $t_{W}$ is the addition time, and $M(t_{W})$ is the concentration of the metal added at $t_{W}$. $M(t^+_{W})$ and $M(t^-_{W})$ are the right and left limits of *M* at $t_{W}$, corresponding to the concentrations of the metal later and before the addition of the waste. In particular, when $t_{W}$ is equal to 0, $M(t_{W})$ represents the initial condition of metal in solid form ($M^0$).

The experimental data were compared with model predictions, adjusting and varying specific kinetic, stoichiometric, and physico-chemical parameters until model results adequately fitted the experimental data. The calibration phase was based on experimental data of glucose degradation, butyric and acetic acid production, cumulative hydrogen generation and manganese trend in the tests. The required initial condition for model calibration was set according to lab-scale experiments and are reported in Table 1. According to the literature^43,46,47^, the common parameters with the ADM1 were selected as base-values due to the similarity of anaerobic digestion and DF processes. Indeed, DF can be seen as an anaerobic digestion process in which the last step of *methanogenesis* is suppressed to produce hydrogen instead of methane. The numerical results were achieved with an original code implemented in MatLab platform. The model was rerun several times by increasing/reducing each parameter, one by one, until the model well reproduced the experimental data. All the values of stoichiometric, kinetic and operating parameters resulting from the calibration phase are reported in Supplementary Table S2. In particular, the parameters related to the leaching process play a fundamental role. Indeed, the leaching inhibition constant $K_{L}$ regulates the metals inhibition function $I_{L}$ (Eq. 49). When the waste is added at the beginning of the process (*IC*1), $I_{L}$ is less than 1 and the DF process is inhibited. The higher is the amount of waste added to the bioreactor, the lower is $I_L$. When the waste is added later (*IC*2), the DF evolves without inhibition and $I_L$ is equal to 1. The dissolution and reduction constants ($k_{d}$ and $k_{r}$) regulate the processes in which the metals are involved. The constant $k_{d}$ is related to the conversion of the metal from the solid to the liquid form, while the subsequent reduction of metals directly depends on $k_{r}$. According to experimental data, manganese was initially dissolved consuming butyric acid. Subsequently, a reduction of the dissolved metal concentration was observed. Noteworthy, the only manganese leaching process was considered in the mathematical model, and butyric acid was exclusively used for the dissolution process. This can represent a limiting assumption for the model, but the main scope of this work was to study the feasibility of the combined DF-leaching process and to develop a mathematical model that can catch the main phenomena.

The biological parameters, $f_{bu,su}$, $f_{pro,su}$, $f_{ac,su}$, $f_{H_2,su}$, $Y_{su}$, $K_{su}$, and $\mu _{max,su}$ were calibrated without considering the inhibition of metals. Indeed, the fraction of hydrogen, butyric, propionic, and acetic acid from sugar ($f_{H_2,su}$, $f_{bu,su}$, $f_{pro,su}$, and $f_{ac,su}$) describe the amount of these products deriving from the glucose conversion. Differently from ADM1, propionic acid was not considered in the model as it was not produced in the bioreactors. Moreover, propionic acid production pathway is usually neglected in DF modeling. Butyrate was the major end-product, followed by acetate. This result is in accordance with the work of Gadhamshetty et al. (2010)^43^, which obtained a quasi null value of $f_{pro,su}$, a $f_{bu,su}$ value grater than $f_{ac,su}$, and a $f_{H_2,su}$ value lower than $20\%$. The other calibrated values of Monod maximum specific uptake rate ($\mu _{max,su}$), yield of biomass ($Y_{su}$), and affinity constant ($K_{su}$) are within the range of values reported in literature^43,47^.

**Figure 1:**
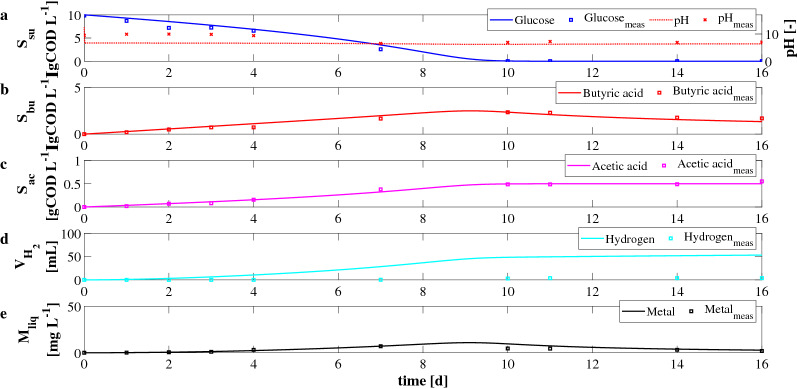
*IC*1—Evolution over time of measured and simulated values of pH (**a**), glucose $S_{su}$ (**a**), butyric acid $S_{bu}$ (**b**), acetic acid $S_{ac}$ (**c**) concentrations, cumulative hydrogen production $V_{H_2}$ (**d**), and concentration of metal in solution $M_{liq}$ (**e**).

The quality of the calibration was evaluated through the determination of performance indexes^48^: the mean absolute error (MAE), including its normalized form (NMAE), the modeling efficiency (ME), the index of agreement (IoA), and the fractional mean bias (FB). Such indexes were frequently used for calibration purposes^49^, and are usually reported as:

52 $$\begin{aligned}&MAE=\frac{\sum _{i=1}^N |P_i - O_i|}{N} \end{aligned}$$

53 $$\begin{aligned}&NMAE=\frac{MAE}{\bar{O}} \end{aligned}$$

54 $$\begin{aligned}&ME=1- \frac{\sum _{i=1}^N \left( P_i - O_i\right) ^2}{\sum _{i=1}^N \left( O_i - {\bar{O}}\right) ^2} \end{aligned}$$

55 $$\begin{aligned}&IoA=1- \frac{\sum _{i=1}^N \left( P_i - O_i\right) ^2}{\sum _{i=1}^N \left( \left| P_i - {\bar{O}} \right| + \left| O_i - {\bar{O}} \right| \right) ^2} \end{aligned}$$

56 $$\begin{aligned}&FB=\frac{{\bar{P}}-{\bar{O}}}{\frac{1}{2}({\bar{P}}+{\bar{O}})} \end{aligned}$$

where *N* is the number of available values; $P_i$ and $O_i$ denote the *i*th predicted value and the *i*th observed value, respectively; ${\bar{P}}$ and ${\bar{O}}$ denote their mean values. The performance indicators are provided in Table 2. The results related to the *IC*1 showed that the *NMAE* and *FB* error indexes were lower than about 14%, and the *ME* and *IoA* error indexes were greater than about 93%, except for $V_{H_2}$ and $M_{liq}$ (Fig. 1). In the case of *IC*2, the *NMAE* and *FB* error indexes were lower than about 11%, while the *ME* and *IoA* error indexes were greater than about 94% for all model variables.

Experimental and model results related to the *IC*1 are reported in Fig. 1. The initial condition of model simulation was characterized by: 10 gCOD L$^{-1}$ of glucose ($S^0_{su}$), 5 gCOD L$^{-1}$ of mixed culture ($X^0_{su}$), and $1\,{\text{g}}$ of E-waste added at $t_{W}=0\,{\text{d}}$. Figure 1 shows that the model was able to fit experimental data related to the biological process. The addition of E-waste negatively affected the DF process in terms of hydrogen and VFAs yields. Indeed, the complete conversion of glucose (Fig. 1a) was achieved in about 10 days, due to a partial inhibition of the E-waste on sugar fermenters $X_{su}$. Consequently, the production of VFAs and hydrogen was observed. The butyric acid (Fig. 1b) initially had an increasing trend, and it reached the maximum predicted value of 2.5 gCOD L$^{-1}$ at $t=9\,{\text{d}}$. Due to the dissolution process, butyric acid consumption occurred in the second part of the experiments. At $t=16\,{\text{d}}$ the measured residual concentration of butyric acid was equal to 1.7 gCOD L$^{-1}$. This result is confirmed by Wang et al. (2019), who reported that butyric acid is the most effective leaching reagent^14^. Similar carboxylic acids, such as acetic and propionic acids, are characterized by a lower efficiency than butyric acid, and their presence has limited effect on the leaching process^50^. The acetic acid (Fig. 1c) showed an increasing trend in all the observation period. The predicted values of both butyric acid and acetic acid at $t=16\,{\text{d}}$ were approximately equal to the measured residual concentrations. The model accurately reproduced experimental data except for the cumulative hydrogen production (Fig. 1d). Its overestimation is probably due to the presence of other chemical compounds contained in the E-waste, as it is well known that hydrogen is a high reactive compound for secondary reactions. Nevertheless, these chemical reactions were not considered in the mathematical model. For this reason, at $t=16\,{\text{d}}$ the predicted value of hydrogen was 53.6 mL, while the measured value was equal to 3.7 mL. As shown in Fig. 1a, it is possible to observe an reasonable fit in the case of pH values. High pH was observed at the beginning of the process due to the waste addition, while the value decreased over time due to acids accumulation. Figure 1e shows the results related to the metal leaching and precipitation processes. The leaching process begun at day 1 and the concentration of the leached manganese reached the maximum predicted value of $10.{8}\,\hbox {mg L}^{-1}$ at $t=9\,{\text{d}}$. Subsequently, a slow decrease was observed and the predicted and the measured concentrations of $2.{7}\,\hbox {mg L}^{-1}$ and $1.9\,\hbox {mg L}^{-1}$ were observed at $t=16\,{\text{d}}$, respectively. Such reduction can be ascribed to precipitation and/or adsorption phenomena observed in previous studies^37^. Nevertheless, the model prediction of $M_{liq}$ was less accurate than other variables due to the uncertainty about all the possible chemical reactions occurring in the bioreactors.

**Figure 2:**
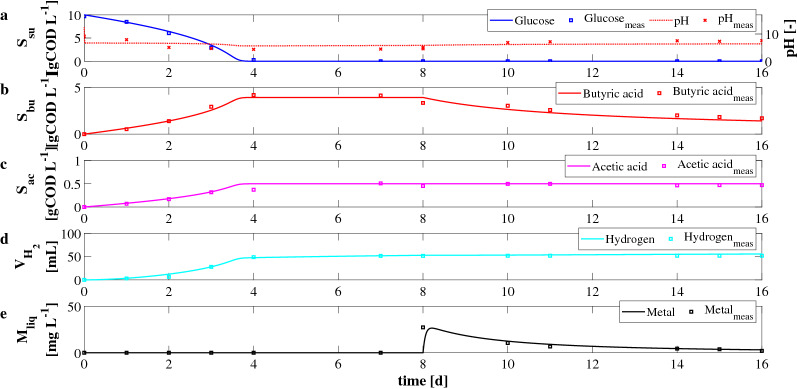
*IC*2—Evolution over time of measured and simulated values of pH (**a**), glucose $S_{su}$ (**a**), butyric acid $S_{bu}$ (**b**), acetic acid $S_{ac}$ (**c**) concentrations, cumulative hydrogen production $V_{H_2}$ (**d**), and concentration of metal in solution $M_{liq}$ (**e**).

Experimental and model results related to the *IC*2 are reported in Fig. 2. In this case, the initial conditions were set to: 10 gCOD L$^{-1}$ of glucose ($S^0_{su}$), 5 gCOD L$^{-1}$ of mixed culture ($X^0_{su}$), and $1\,{\text{g}}$ of E-waste added once the produced hydrogen by the DF process achieved a constant value ($t_{W}=8\,{\text{d}}$). In this case, a good agreement between predicted and experimental data was observed. Obviously, the biological process was not negatively affected by metal concentration and the complete consumption of glucose (Fig. 2a) was obtained after about 4 days. Consequently, the production of butyric acid (Fig. 2b), acetic acid (Fig. 2c), and hydrogen (Fig. 2d) was observed. The predicted maximum values of butyric and acetic acids were 3.9 gCOD L$^{-1}$ and 0.5 gCOD L$^{-1}$, respectively. Once the waste was added to the bioreactors, $S_{bu}$ decreased over time due to the leaching process. The production and consumption phases are clearly separated and take place consecutively. It means that butyric acid initially produced during the fermentation, and it was consumed when the leaching process begun. However, a residual butyric acid concentration was observed. The predicted and measured residual concentration at $t=16\,{\text{d}}$ were equal to 1.5 gCOD L$^{-1}$ and 1.7 gCOD L$^{-1}$. The cumulative predicted and measured hydrogen production after 16 days were equal to 55 mL and 52 mL, respectively. As shown in Fig. 2a, pH decreased during the fermentation due to acids production. In Fig. 2e the results related to the metal leaching process are reported. Once the E-waste was added to bioreactors, the butyric acid and the solid manganese were rapidly involved in the leaching process. The maximum value of manganese in the solution was observed after a few hours of simulation, and it was equal to 26.5 mg L$^{-1}$. Subsequently, the dissolved manganese concentration decreased due to the precipitation/adsorption process, and it reached a residual value according to the experimental evidence (predicted and measured concentration of 3 mg L$^{-1}$ and 2 mg L$^{-1}$, respectively).

**Table 1:** Initial conditions and operating parameters used for model calibration.

Parameter	Definition	Unit	IC1	IC2
$S^0_{su}$	Initial concentration of glucose	kgCOD m$^{-3}$	10	10
$S^0_{bu}$	Initial concentration of butyric acid	kgCOD m$^{-3}$	0	0
$S^0_{ac}$	Initial concentration of acetic acid	kgCOD m$^{-3}$	0	0
$S^0_{H_2}$	Initial concentration of hydrogen	kgCOD m$^{-3}$	0	0
$S^0_{IC}$	Initial concentration of inorganic carbon	kmol m$^{-3}$	0.1	0.1
$S^0_{IN}$	Initial concentration of inorganic nitrogen	kmol m$^{-3}$	0.06	0.06
$S^0_{bu^-}$	Initial concentration of butyrate	kgCOD m$^{-3}$	0	0
$S^0_{ac^-}$	Initial concentration of acetate	kgCOD m$^{-3}$	0	0
$S^0_{HCO_3^-}$	Initial concentration of bicarbonate	kmol m$^{-3}$	0	0
$S^0_{NH_3}$	Initial concentration of ammonia	kmol m$^{-3}$	0	0
$X^0_{su}$	Initial concentration of sugar fermenters	kgCOD m$^{-3}$	5	5
$S^0_{gas,H_2}$	Initial concentration of hydrogen gas	kgCOD m$^{-3}$	0	0
$S^0_{gas,CO_2}$	Initial concentration of carbon dioxide gas	kmol m$^{-3}$	0	0
$M_{liq}^0$	Initial concentration of metal in liquid form	kg m$^{-3}$	0	0
$M(t_{W})$	Metal concentration added to the bioreactor at $t_{W}$	kg m$^{-3}$	6.5	6.5
$t_{W}$	Addition time of E-waste	d	0	8

**Table 2:** Performance indicators.

IC1				
Variable	NMAE	ME	IoA	FB
$S_{su}$	0.0929	0.9772	0.9946	0.0862
$S_{bu}$	0.1445	0.9279	0.9802	0.0178
$S_{ac}$	0.0691	0.9842	0.9959	-0.0088
$V_{H_2}$	16.479	-338.107	0.1277	1.7835
$M_{liq}$	0.4213	0.2462	0.8734	0.3092

IC2				
Variable	NMAE	ME	IoA	FB
$S_{su}$	0.0593	0.9958	0.9990	0.0301
$S_{bu}$	0.1146	0.9397	0.9846	-0.0654
$S_{ac}$	0.0726	0.9376	0.9851	0.0665
$V_{H_2}$	0.0507	0.9850	0.9963	0.0448
$M_{liq}$	0.1161	0.9932	0.9958	0.0837

## Numerical studies and results

Further numerical simulations were performed to study the removal efficiency of metals from E-waste by adopting the DF process. To optimize the integrated DF-leaching process, three fundamental aspects were investigated: (i) the effect of metal inhibition on the biological process; (ii) the effect of *F*/*M* ratio on the bioconversion of sugars; and (iii) the effect of butyric acid and metal concentration on the leaching efficiency. To this aim, four numerical studies were implemented:

- *NS*1 examines the effect of the initial metal concentration, $M^0$, on the microbial activity;
- *NS*2 and *NS*3 investigate the effect of the *F*/*M* ratio variation, $S^0_{su}/X^0_{su}$, on the DF process and on the leaching efficiency;
- *NS*4 explores the effect of E-waste concentration, $M(t_{W})$, on the metal removal efficiency.

To mitigate the negative effect of metals on the biological process, the strategy adopted in the numerical study *NS*1 was to change the initial metal concentration in the bioreactor. In this case, the results are presented in the timescale from day 0 to day 16. As shown in the experimental results, the concentration of the dissolved metal decreased in the last part of the experiments. This reduction was due to the precipitation of the metal in the bioreactor or to the adsorption of the metal on solid components involved in the process^18,37^. Since the recovery of metals can be easier achieved from the leachate solution^9^, such reduction makes the leaching process inefficient^14^. This naturally leads to consider a sequential batch reactor or two separate reactors, to avoid inhibition effects of metals during DF and to maximize metal recovery using the clarified DF effluent as leaching agent. For this reason, the strategy adopted in the numerical studies *NS*2, *NS*3, and *NS*4 consisted in separating the biological process and the leaching reaction in two different consecutive reactors. The first bioreactor contains the microbial inoculum, and, it is fed with sugar and inoculum, allowing for the biological process evolution. Once the DF is completely developed, the effluent, rich in VFAs, is used for the leaching process in the second reactor. To avoid precipitation and/or adsorption phenomena and to maximize the metal recovery, the treatment time of the waste in the second reactor was fixed to 24 h^37^. Indeed, the dissolution reaction can be considered significantly faster than the precipitation/adsorption process. In this condition, it can be assumed that a negligible precipitation/adsorption of the metal occurs. With these assumptions, the precipitation process was neglected ($\rho_{M,2}=0$) in numerical studies *NS*2, *NS*3, and *NS*4. The results related to the biological process are presented in the timescale from day 0 to day 8, and the results related to the leaching process are presented in the same Figure in the timescale from day 8 to day 9. Successively, ad-hoc and controlled processes can be further used for the final recovery of metals from the DF effluent-leachate solution, such as electrochemical, precipitation, or solvent extraction techniques^6,7,9,37^.

The initial conditions $S^0_{su}$, $X^0_{su}$, $M^0$, and $M(t_{W})$, were varied in numerical studies. Their values have been highlighted below for each numerical study. The values of kinetic, stoichiometric and leaching parameters were derived from the calibration phase. All the values of stoichiometric, kinetic and operating parameters are summarized in the Supplementary Table S2.

### NS1—Effects of metal inhibition

As aforementioned, metals are able to inhibit the fermentative process evolution due to their toxic effect. The experimental data clearly showed this inhibition phenomenon when the waste was added at the beginning of the experiments. In this context, a numerical study *NS*1 was performed to study the metal inhibition effect on the biological process. Four numerical simulations were carried out with different initial concentrations of metal $M^0$ (6.5, 3, 1.5, and 0.5 g L$^{-1}$). The same initial concentration of glucose and sugar fermenters were used: $S^0_{su} =10$ gCOD L$^{-1}$ and $X^0_{su} =5$ gCOD L$^{-1}$. Consequently, the value of *F*/*M* ratio used in the numerical simulations was $2\,{gCOD}_{substrate} \ {gVS}^{-1}_{inoculum}$. The remaining initial conditions set for this numerical study were the same used in the calibration phase.

**Figure 3:**
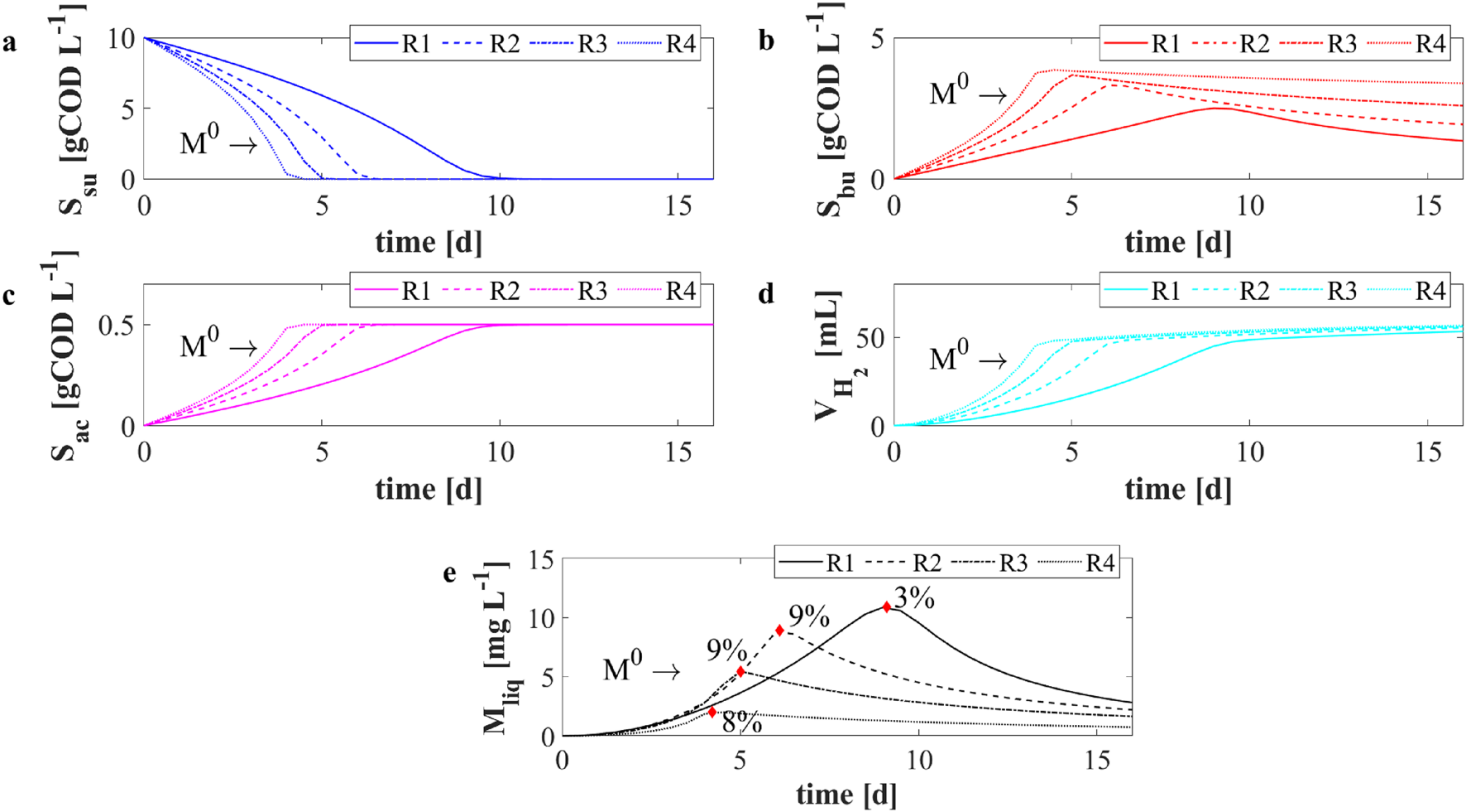
*NS*1—Evolution over time of simulated values of glucose $S_{su}$ (**a**), butyric acid $S_{bu}$ (**b**), acetic acid $S_{ac}$ (**c**) concentrations, cumulative hydrogen production $V_{H_2}$ (**d**), and concentration of metal in solution $M_{liq}$ (**e**) for different initial concentrations of metal $M^0$. *R*1: $M^0={6.5}\,\hbox {g L}^{-1}$; *R*2: $M^0={3}\,\hbox {g L}^{-1}$; *R*3: $M^0={1.5}\,\hbox {g L}^{-1}$; *R*4: $M^0=0.5\,\hbox {g L}^{-1}$. Initial concentration of sugar and sugar fermenters: $S^0_{su} =10\,\hbox {gCOD L}^{-1}$ and $X^0_{su} =5\,\hbox {gCOD L}^{-1}$. Red rhombus represents the maximum removal efficiency.

The model results of *NS*1 are shown in Fig. 3. The time required for the complete glucose degradation decreased from 10 to 4 days (Fig. 3a) when the initial concentration of metal decreased from 6.5 to 0.5 g L$^{-1}$. Clearly, this behaviour can be attributed to the metal inhibition function, which is inversely proportional to the concentration of the metal in solid form. Consequently, the productions rates of VFAs (Fig. 3b and c) and hydrogen (Fig. 3d) were faster for low values of $M^0$. All DF end-products (except for the butyric acid) approximately reached the same concentration when the residual concentrations of glucose were close to zero. The butyric acid (Fig. 3b), which is directly involved in the leaching reactions, reached its maximum value when the lowest concentration of metal was fed to the reactor (*R*4). Of course, in this condition the residual concentration of butyric acid was higher due to the limited presence of the metal. Finally, when a smaller amount of E-waste was added to the bioreactor, the inhibition effect was less intense and the butyric acid was faster produced. Figure 3e shows the results related to the metal dissolution. As expected, the leaching process led to a higher metal concentration in dissolved form in *R*1 but in a longer time. Nevertheless, the maximum removal efficiency (Fig. 3e—red rhombus) obtained in each simulation was lower than 9%. When the initial concentration of metal in solid form was equal to 6.5 g L$^{-1}$ (*R*1), the dissolved metal concentration initially increased with the highest rate. Nevertheless, around day 3, a rapid increment was observed in the cases of initial solid metal of *R*2 and *R*3. Indeed, the leaching rate depends linearly on the metal concentration and quadratically on butyric acid concentration. At the beginning of the numerical experiments, the butyric acid concentration was low as the complete glucose degradation was not reached. Successively, the butyric acid increased, allowing for the dissolution of the metal.

### NS2 and NS3—Effects of F/M ratio on DF process

The *F*/*M* ratio plays a fundamental role in the dark fermentation, especially when the process is devoted to hydrogen production^19^. To avoid methane generation and maximize hydrogen production, high *F*/*M* ratios (generally higher than 1) are used for the DF process. For this reason, a numerical study *NS*2 was conducted to investigate the effect of the *F*/*M* ratio ($S^0_{su}/X^0_{su}$ in the mathematical model) on the DF evolution. Specifically, 10 simulations were carried out with different initial concentrations of glucose $S^0_{su}$ (5, 10, 15, 20, 25, 30, 35, 40, 45, and 50 gCOD L$^{-1}$). The same initial condition of sugar fermenters and metal concentration were used: $X^0_{su} =5\,\hbox {gCOD L}^{-1}$ and $M ={6.5}\,\hbox {g L}^{-1}$. Consequently, the value of *F*/*M* ratio used in the numerical simulations varied from 1 to 10 gCOD$_\mathrm{substrate}\ gVS^{-1}_{inoculum}$. The remaining initial conditions set for this numerical study were the same used in the calibration phase.

**Figure 4:**
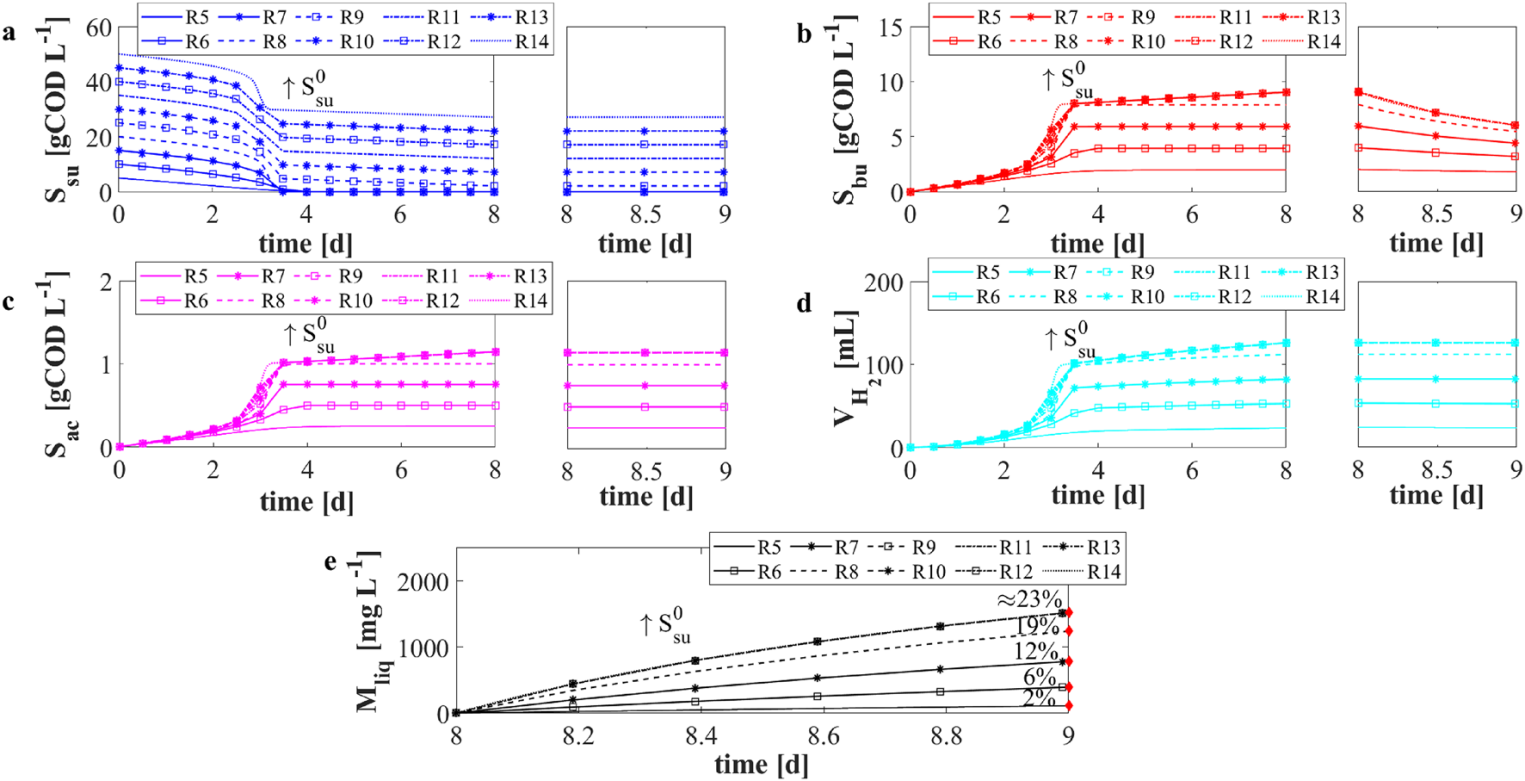
*NS*2—Evolution over time of simulated values of glucose $S_{su}$ (**a**), butyric acid $S_{bu}$ (**b**), acetic acid $S_{ac}$ (**c**) concentrations, cumulative hydrogen production $V_{H_2}$ (**d**), and concentration of metal in solution $M_{liq}$ (**e**) for different initial concentrations of sugar $S^0_{su}$. *R*5: $S^0_{su}=5\,\hbox {gCOD L}^{-1}$; *R*6: $S^0_{su}=10\,\hbox {gCOD L}^{-1}$; *R*7: $S^0_{su}=15\,\hbox {gCOD L}^{-1}$; *R*8: $S^0_{su}=20\,\hbox {gCOD L}^{-1}$; *R*9: $S^0_{su}=25\,\hbox {gCOD L}^{-1}$; *R*10: $S^0_{su}=30\,\hbox {gCOD L}^{-1}$; *R*11: $S^0_{su}=35\,\hbox {gCOD L}^{-1}$; *R*12: $S^0_{su}=40\,\hbox {gCOD L}^{-1}$; *R*13: $S^0_{su}=45\,\hbox {gCOD L}^{-1}$; *R*14: $S^0_{su}=50\,\hbox {gCOD L}^{-1}$. Initial concentration of sugar fermenters and metal concentration: $X^0_{su} =5\,\hbox {gCOD L}^{-1}$ and $M={6.5}\,\hbox {g L}^{-1}$. Red rhombus represents the removal efficiency after 24 h.

The results of *NS*2 are summarized in Fig. 4. When *F*/*M* ratio increased from 1 to 4 $gCOD_{substrate} \ gVS^{-1}_{inoculum}$, all the biological process rates increased. Thus, glucose (Fig. 4a) was completely consumed at day 4, and high concentrations of VFAs (Figs. 4b and 4c) and hydrogen (Fig. 4d) were obtained at the end of the biological process. Nevertheless, for *F*/*M* ratios greater than 4 the process was inhibited, and the glucose was not completely degraded. Indeed, when the initial concentration of glucose was higher than 20 gCOD L$^{-1}$, the biological environment was characterized by inhibiting pH levels due to high VFAs concentrations. By setting the initial glucose concentration $S^0_{su}$ from 25 to 50 gCOD L$^{-1}$, the residual concentration of glucose at $t=8\,{\text{d}}$ increased, and similar VFAs and hydrogen productions were observed. From day 8 to 9, a second reactor with the same volume was considered, and the composition of the liquid environment was dictated by the DF effluent composition in terms of dissolved compounds. The butyric acid consumption was immediately observed due to the metal addition (Fig. 4b). The higher was the butyric acid concentration at the end of the DF stage (from day 0 to day 8), the faster was its consumption in the second stage (from day 8 to day 9). Indeed, the leaching rate quadratically depends on acid concentration. The trend of dissolved metal is reported in Fig. 4e. As explained above, the removal efficiency was computed after 24 h (Fig. 4e—red rhombus). The removal efficiency after 24 h reached higher values when increasing the *F*/*M* ratio from 1 to 4. When the initial glucose concentration was set from 20 to 25 gCOD L$^{-1}$, the removal efficiency increased about 5%. When values of *F* greater than 25 gCOD L$^{-1}$ were adopted, the concentration of the leached metal was constant, as the same concentration of butyric acid was predicted in the reactor. This result is confirmed by several studies^13,37,51^, showing that the increase of acidic concentrations over specific thresholds does not affect the leaching efficiency in the case of both organic and inorganic acids. In conclusion, the optimal *F*/*M* ratio $4 \ gCOD_{substrate} \ gVS^{-1}_{inoculum}$ was obtained.

To improve VFAs and hydrogen production, and consequently the metal removal efficiency, the numerical study *NS*3 was performed. Again 10 different numerical experiments were carried out with different initial conditions of glucose $S^0_{su}$ (5, 10, 15, 20, 25, 30, 35, 40, 45, and 50 gCOD L$^{-1}$), with the same metal concentration ($M ={6.5}\,\hbox {g L}^{-1}$), but with an initial sugar fermenters concentration $X^0_{su}$ equal to 10 gCOD L$^{-1}$. This choice was aimed at decreasing the degradation and production time of the biological compartment to enhance the metal recovery efficiency. The initial condition of inorganic carbon and inorganic nitrogen were doubled as well with respect to the numerical study *NS*2. The *F*/*M* ratios used in this numerical study varied from 0.5 to 5 $gCOD_{substrate} \ gVS^{-1}_{inoculum}$. The remaining initial conditions set for this numerical study were the same used in the calibration phase.

**Figure 5:**
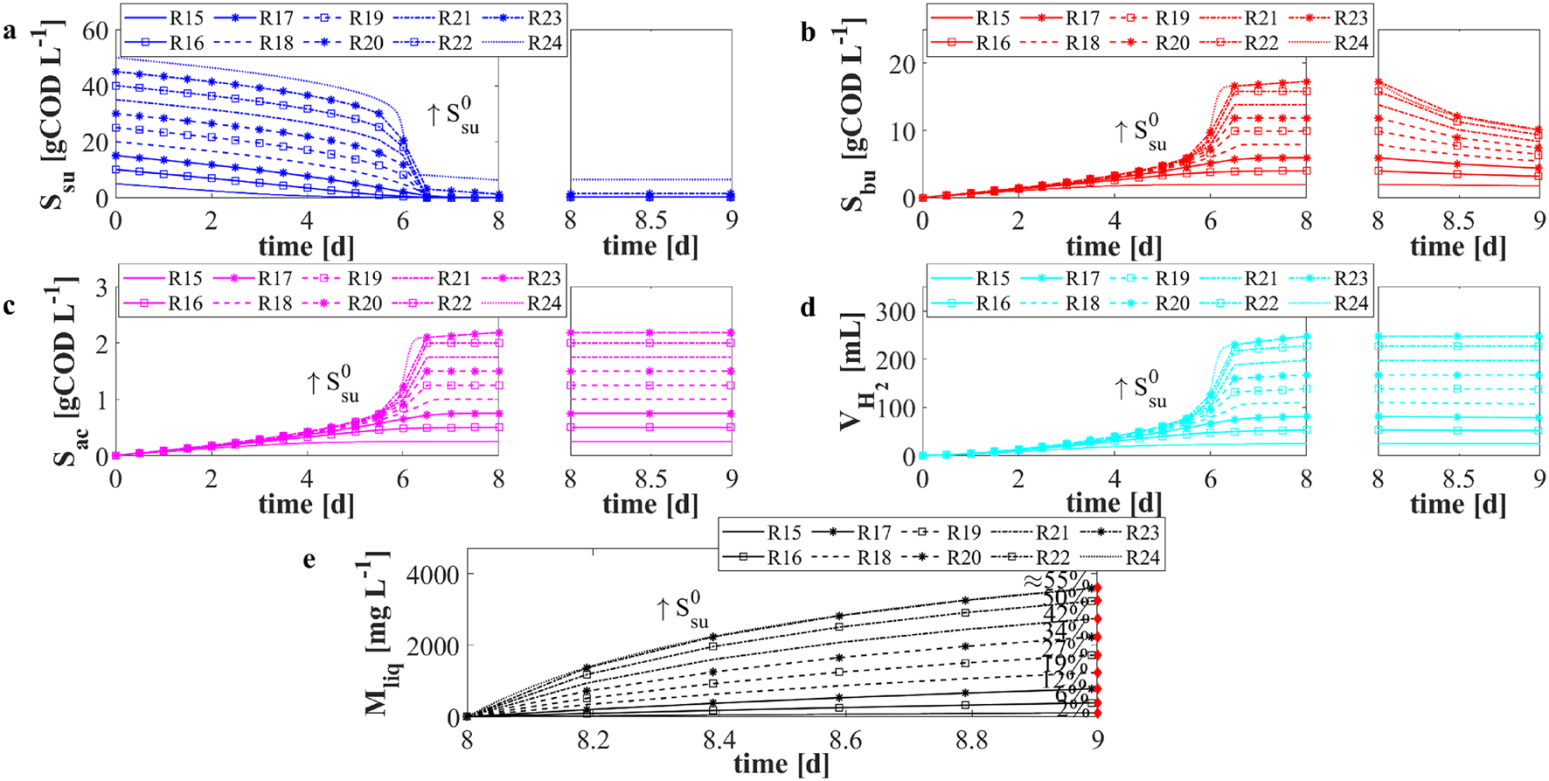
*NS*3—Evolution over time of simulated values of glucose $S_{su}$ (**a**), butyric acid $S_{bu}$ (**b**), acetic acid $S_{ac}$ (**c**) concentrations, cumulative hydrogen production $V_{H_2}$ (**d**), and concentration of metal in solution $M_{liq}$ (**e**) for different initial concentrations of sugar $S^0_{su}$. *R*15: $S^0_{su}=5\,\hbox {gCOD L}^{-1}$; *R*16: $S^0_{su}=10\,\hbox {gCOD L}^{-1}$; *R*17: $S^0_{su}=15\,\hbox {gCOD L}^{-1}$; *R*18: $S^0_{su}=20\,\hbox {gCOD L}^{-1}$; *R*19: $S^0_{su}=25\,\hbox {gCOD L}^{-1}$; *R*20: $S^0_{su}=30\,\hbox {gCOD L}^{-1}$; *R*21: $S^0_{su}=35\,\hbox {gCOD L}^{-1}$; *R*22: $S^0_{su}=40\,\hbox {gCOD L}^{-1}$; *R*23: $S^0_{su}=45\,\hbox {gCOD L}^{-1}$; *R*24: $S^0_{su}=50\,\hbox {gCOD L}^{-1}$. Initial concentration of sugar fermenters and metal concentration: $X^0_{su}=10\,\hbox {gCOD L}^{-1}$ and $M={6.5}\,\hbox {g L}^{-1}$. Red rhombus represents the removal efficiency after 24 h.

The results related to *NS*3 are reported in Fig. 5. These confirmed that the increase of *F*/*M* ratio favoured the biological process evolution. Nevertheless, when *F*/*M* ratio greater than 4 (i.e. $S^0_{su}$ greater than $40\,\hbox {gCOD L}^{-1}$) were used, the process was inhibited by the pH level, and the glucose was not completely consumed by microorganisms. Thus, a residual concentration of glucose was observed, but the same amount of VFAs and hydrogen were produced. In the second reactor (day 8), butyric acid was consumed (Fig. 5b), and contextually the concentration of metal in solution quickly increased (Fig. 5e). By increasing the initial glucose concentration $S^0_{su}$, the predicted concentration of butyric acid increased. Consequently, the dissolution rate was faster and the removal efficiency after 24 h increased (Fig. 5e—red rhombus). In accordance with the previous numerical study, *F*/*M* ratios greater than 4 did not lead to a beneficial effect on the leaching efficiency. The optimal *F*/*M* ratio was again 4 in terms of VFAs and hydrogen productions. In addition, using 40 and 10 gCOD L$^{-1}$ of glucose and digestate, respectively, the leached metal concentration in the solution significantly increased, due to the increased butyric acid concentration produced in the first stage. The obtained removal efficiency was about $50\%$. This result is in accordance with experimental evidences in which the leaching process was catalyzed by organic acids^14,37^. Nevertheless, other experimental works reported a leaching efficiency of about 90%^9,13^. This suggests that the leaching process strictly depends on the type of metal and organic acid investigated. However, the obtained removal efficiency is relatively low if compared with data related to processes performed with inorganic acids used as leaching agents^16,51^.

### NS4—Effects of metal concentration on leaching process

Many experimental works focused on metals recovery efficiency using different ratios between concentrations of metals and organic acids^9,18^. To identify the optimum leaching conditions, the numerical study *NS*4 was performed. Six numerical simulations were carried out with different concentrations of solid metal *M* (10, 6.5, 3, 1, 0.5, and 0.1 g L$^{-1}$). The optimal initial concentrations of glucose and sugar fermenters were used: $S^0_{su} =40\,\hbox {gCOD L}^{-1}$ and $X^0_{su} =10\,\hbox {gCOD L}^{-1}$. Consequently, the value of *F*/*M* ratio used in the numerical simulations was set to $4 \ gCOD_{substrate}\,gVS^{-1}_{inoculum}$. The remaining initial conditions set for this numerical study were the same used in the calibration phase.

**Figure 6:**
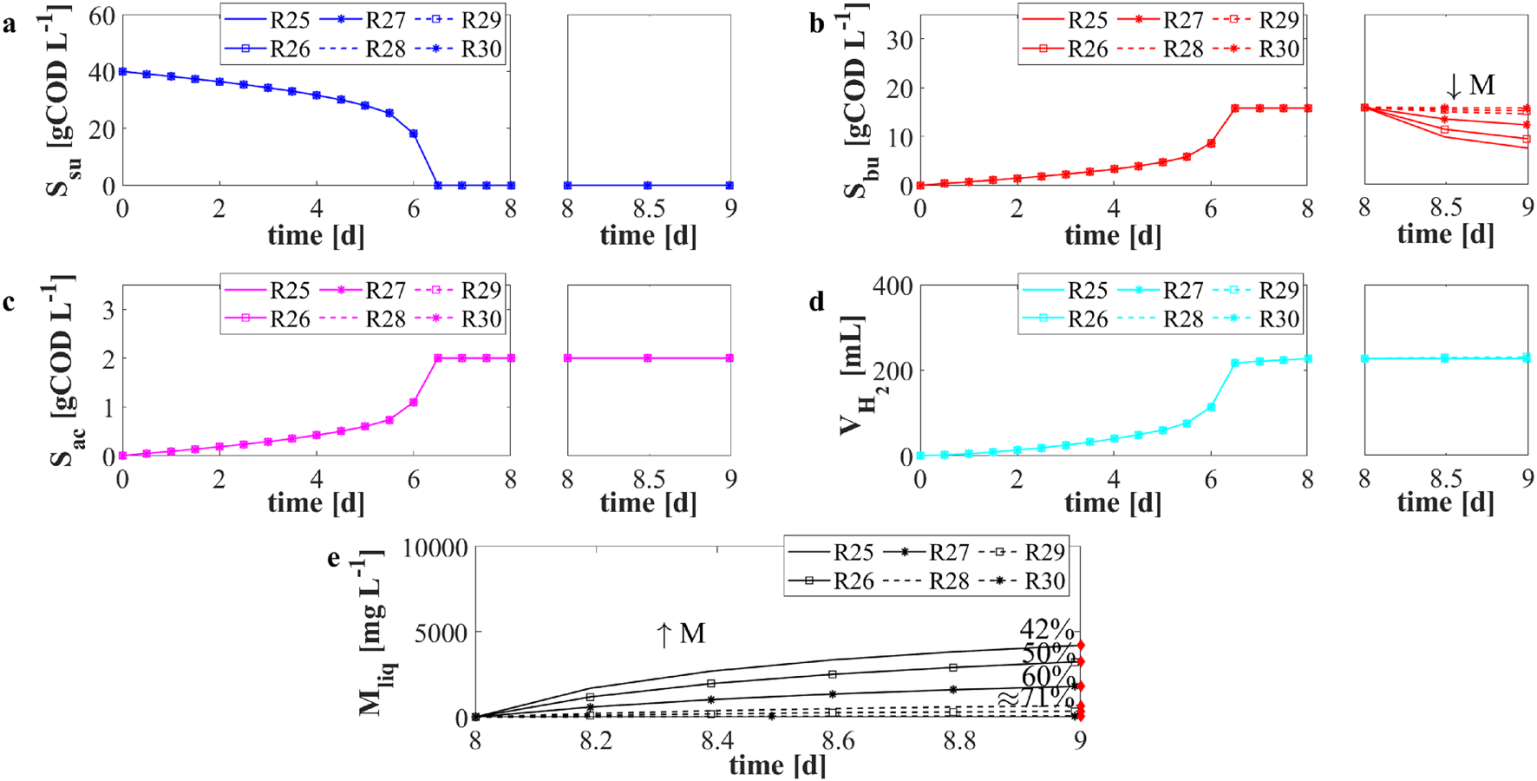
*NS*4—Evolution over time of simulated values of glucose $S_{su}$ (**a**), butyric acid $S_{bu}$ (**b**), acetic acid $S_{ac}$ (**c**) concentrations, cumulative hydrogen production $V_{H_2}$ (**d**), and concentration of metal in solution $M_{liq}$ (**e**) for different concentrations of metal $M(t_W)$. *R*25: $M=10\,\hbox {g L}^{-1}$; *R*26: $M={6.5}\,\hbox {g L}^{-1}$; *R*27: $M={3}\,\hbox {g L}^{-1}$; *R*28: $M=1\,\hbox {g L}^{-1}$; *R*29: $M=0.5\,\hbox {g L}^{-1}$; *R*30: $M=0.1\,\hbox {g L}^{-1}$. Initial concentration of sugar and sugar fermenters: $S^0_{su} =40\,\hbox {gCOD L}^{-1}$ and $X^0_{su} =10\,\hbox {gCOD L}^{-1}$. Red rhombus represents the removal efficiency after 24 h.

*NS*4 results are shown in Fig. 6. In the DF phase, all simulations gave the same results in terms of glucose, VFAs and hydrogen, since a common initial condition related to the biological process was used. Once the E-waste was added to the DF effluent, different butyric acid trends (Fig. 6b) were observed. Increasing the amount of the waste, its consumption was faster and its residual concentration at $t=9\,{\text{d}}$ was lower than those obtained in all the other cases. The trend of the dissolved metal is reported in Fig. 6e. Similarly, when the metal concentration in solid form increased, the metal dissolution was faster, but the leaching efficiency at 24 h (Fig. 6e—red rhombus) decreased. The numerical results showed that the leaching efficiency decreases when an increasing metal concentration and a fixed acid amount are considered. This confirmed the experimental evidences achieved in the case of organic and inorganic acids^13,15^. Obviously, this result suggests that the leaching process is even more rapid, although less efficient, when a higher amount of the waste is added. This leads to conclude that the butyric acid is not a limiting factor, and a longer time is required to leach a greater amount of E-waste.

## Conclusions

A mathematical model able to account for the biological conversion and physico-chemical phenomena involved in the DF and the leaching processes was presented. The model was successfully calibrated with ad-hoc DF experiments carried out using a synthetic solution of glucose, digestate and spent button batteries. Further numerical studies were presented to investigate the optimal conditions to favour the biological conversion of glucose and the leaching process. Specifically, the inhibition of the DF process due to the metal concentration, the effect of the *F*/*M* ratio, and the removal efficiency of the leaching process were investigated by using the model as an experimental tool for the development of the processes in different conditions. In conclusions, the model gave valuable information in terms of: the organic acid concentration required for the leaching process, the amount of leached metal, and the time required for the dissolution process with respect to the amount of E-waste treated in the reactor. Some significant aspects should be further investigated. Specific experimental studies focused on the leaching process of specific metals or metals mixtures in anaerobic environments, and on the effect of leaching by-products on the DF process are still required. The proposed mathematical model could be further improved with additional physico-chemical processes, involving the chemical compounds contained in the E-waste. Indeed, different leaching reactions of other metals contained in the E-waste can be further included. However, the metal recovery efficiency and the residence time can be optimized to study the sustainability of the integrated DF-leaching process at a larger scale.

## Supplementary Information

Supplementary Information.
