# Supplementary material for: Mathematical modeling of metal recovery from E-waste using a dark-fermentation-leaching process
Source: Sci Rep. 2022 Mar 11;12:4274. doi: 10.1038/s41598-022-08106-2 (PMC8917181; doi:10.1038/s41598-022-08106-2)
Supplement: Supplementary file 1 — Supplementary Information. [file 41598_2022_8106_MOESM1_ESM.pdf]

Mathematical Modeling of metal recovery from E-waste using a Dark-Fermentation-Leaching process

Fabiana Russo[1,*,+], Vincenzo Luongo[1,+], Maria Rosaria Mattei[1,+], and Luigi Frunzo[1,+]

[1]University of Naples “Federico II”, Department of Mathematics and Applications “R. Caccioppoli”, via Cintia 1, Monte S. Angelo, 80126, Naples, Italy

[*]fabiana.russo@unina.it
+these authors contributed equally to this work

Experimental campaigns

The experimental campaign was carried out using serum bottles with a volume of 120 mL for DF-leaching experiments. The reactors were immersed in a thermostatic bath at $35°C \pm 1°C$ to ensure mesophilic conditions. The microbial inoculum was obtained from a real-scale anaerobic digestion plant operating the bioconversion of buffalo manure. According to Standard Methods[1], the anaerobic digestate was characterized in terms of Total Solids (TS) and Volatile Solids (VS) to estimate their organic content prior to start the experiments. In particular, the VS content of the digestate was $70.67\ gCOD\ L^{-1}$. The inoculum was thermally pretreated for $1\ h$ at $105°C$ to ensure methanogenesis inhibition[2]. The working volume of the serum bottles was set to $70\ mL$ and was constituted by:

- $5\ mL$ of thermally pretreated inoculum;
- $60\ mL$ of distilled water;
- $5\ mL$ of glucose solution ($141.34\ gCOD\ L^{-1}$);
- $1\ g$ of waste extracted from spent batteries.

The Food/Microorganisms ratio was close to $2\ gCOD_{substrate}\ gVS_{inoculum}^{-1}$ to inhibit metabolic activities of hydrogen consumers and to ensure DF process evolution[2]. Spent button lithium-ion batteries (LIBs) were used as E-waste to investigate the leaching process of metals. LIBs are a common E-waste as they are extensively employed in many anthropic activities due to their superior performance, such as high working voltage, high energy density, small size, low self-discharge rate, and long life-cycle[3]. LIBs contain high concentrations of lithium (Li), cobalt (Co), nickel (Ni), manganese (Mn) and aluminum (Al)[3-5]. In particular, spent button batteries were chosen for the experimental campaign as the internal part is already in powder form, no crushing process is required, and they can be manually disassembled to separate the plastic elements and metallic shells. According to Russo et al. (2022)[6], the overall content of metals in the tested E-waste was evaluated. The internal part of batteries was mineralized with an aqua-regia solution in a START-D microwave oven (Milestone, USA). Mineralized samples were opportunely diluted, filtered at 0,45 μm through cellulose acetate filter and finally analyzed through ICP-MS (PerkinElmer Nexion 350, USA) operating in dual detector mode. Due to its high concentration, Mn content was evaluated through atomic adsorption spectrometry (AAS) using a Varian Model 55B SpectrAA (F-AAS). The detected weight percentage of Mn was equal to 45%, while the results of ICP-MS chemical analysis of the waste are summarized in Supplementary Table S1. The bioreactors were hermetically closed by using specific metal/rubber caps to ensure sampling procedures and anaerobic conditions. Different experimental tests were carried out (Supplementary Figure S3), using the same amount of waste and changing the E-waste addition time: i) by adding the waste at the beginning of the biological process, and ii) with waste addition at the end of the hydrogen production phase.

During the experiments, liquid and gas samples were taken every day from each bioreactor. The extracted liquid and gaseous samples were characterized in terms of glucose and organic acids (OAs) concentrations, and hydrogen production, respectively. In addition, manganese concentration in the liquid phase was evaluated. The manganese concentration in the solution was quantified through atomic adsorption spectrometry (AAS) using a Varian Model 55B SpectrAA (F-AAS). The glucose and OAs concentrations were evaluated by high-pressure liquid chromatography (HPLC), using an LC 25 Chromatography Oven (Dionex, Sunnyvale, CA, USA) equipped with an Organic Acids column (Metrohom, Herisau, Switzerland) and a 340U UV detector (Dionex, Sunnyvale, USA). Temperature and pH were measured with analytic probes.

The hydrogen production was measured using a volumetric method. For gas sampling, the bioreactors were connected with a gas measurement system. The gas was forced to pass into a bottle filled with HCl solution to trap the produced carbon dioxide before hydrogen evaluation. This procedure allowed for the determination of the produced hydrogen volume. Biogas composition was characterized by gas chromatographic analysis conducted using a Varian Star 3400 gas chromatograph equipped with a ShinCarbon ST 80/100 column and a thermal conductivity detector. Argon as used as gas carrier for gaseous samples.

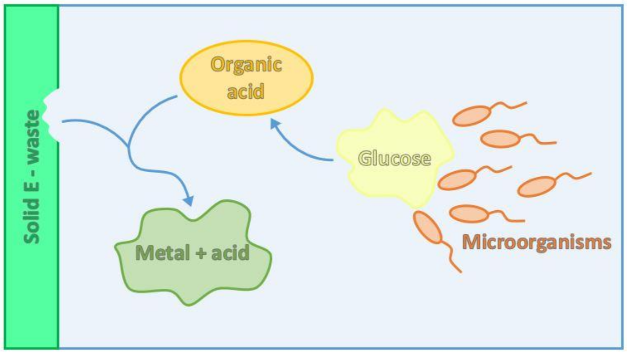

Figure S1. Scheme of dark fermentation and leaching processes

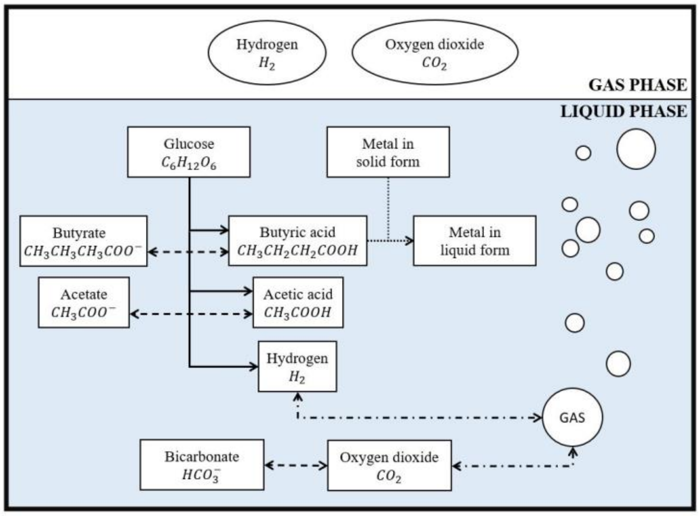

Figure S2. Conversion scheme of the dark fermentation and the leaching process (adapted and modified from Batstone et al. (2002)[7]). Solid arrows: biological reactions; dashed arrows: acid-base equilibrium; dash-dot arrows: liquid-gas transfer; dotted arrows: leaching process. Biochemical and leaching reactions are modeled as irreversible processes, while physico-chemical reactions (acid-base and liquid-gas equilibria) are implemented as reversible processes. Glucose: S_{su}; butyric acid: S_{bu}; butyrate: $S_{bu}{}^{-}$; acetic acid: S_{ac}; acetate: $S_{ac}{}^{-}$; hydrogen: S_{H_2}; hydrogen gas: S_{gas,H_2}; oxygen dioxide: S_{CO_2}; bicarbonate: $S_{HCO_3{}^-}$; oxygen dioxide gas: S_{gas,CO_2}; metal in solid form: M; metal in liquid form: M_{liq}.

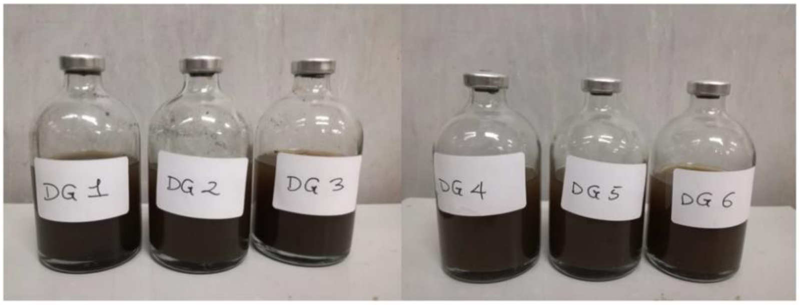

Figure S3. Experimental campaigns bioreactors.

Metal	Content (wt%)
Al	0.009414
As	0.000038
B	0.000489
Ba	0.000643
Be	0.000001
Bi	0.000036
Ca	0.050509
Cd	0.000003
Co	0.000080
Cr	0.005796
Cu	0.000795
Fe	0.105971
Ga	0.000023
Hg	0.000813
In	0.000000
K	0.034315
Li	9.448186
Mg	0.038388
Mo	0.000093
Na	0.127137
Ni	0.005718
Pb	0.000207
Sb	0.000022
Se	0.000140
Si	0.183181
Sn	0.000018
Sr	0.000893
Te	0.000019
Tl	0.000177
V	0.000032
Zn	0.001302

Table S1. Results of ICP-MS chemical analysis of the internal part of spent button LIBs.

Parameter	Definition	Unit	Value	Reference
V	Reactor volume	mL	120	Experimental value
V_{liq}	Reactor volume	mL	70	Experimental value
V_{gas}	Reactor volume	mL	50	Experimental value
Y_{su}	X_{su} yield on S_{su}	$kgCOD\ kgCOD^{-1}$	0.5	Calibrated value
$f_{bu,su}$	fraction of S_{bu} from S_{su}	$kgCOD\ kgCOD^{-1}$	0.79	Calibrated value
$f_{ac,su}$	fraction of S_{ac} from S_{su}	$kgCOD\ kgCOD^{-1}$	0.10	Calibrated value
$f_{H_2,su}$	fraction of S_{H_2} from S_{su}	$kgCOD\ kgCOD^{-1}$	0.11	Calibrated value
C_{su}	Carbon content of S_{su}	$kmoleC\ kgCOD^{-1}$	0.0313	Batstone et al. (2002)[7]
C_{bu}	Carbon content of S_{bu}	$kmoleC\ kgCOD^{-1}$	0.025	Batstone et al. (2002)[7]
C_{ac}	Carbon content of S_{ac}	$kmoleC\ kgCOD^{-1}$	0.0313	Batstone et al. (2002)[7]
C_{biom}	Carbon content of biomass	$kmoleC\ kgCOD^{-1}$	0.0313	Batstone et al. (2002)[7]
N_{biom}	Nitrogen content of biomass	$kmoleN\ kgCOD^{-1}$	0.00625	Batstone et al. (2002)[7]
$\mu_{max,su}$	Monod maximum specific uptake rate of X_{su}	d^{-1}	2	Calibrated value
K_{su}	S_{su} affinity constant for X_{su}	$kgCOD\ m^{-3}$	1.5	Calibrated value
$k_{dex,X_{su}}$	Decay-inactivation rate for X_{su}	d^{-1}	0.02	Batstone et al. (2002)[7]
k_d	Dissolution constant	$kgCOD^2\ m^{-6}\ d^{-1}$	0.005	Calibrated value
k_r	Reduction constant	d^{-1}	17	Calibrated value
$K_{A/B,bu}$	S_{bu} acid-base kinetic parameter	$m^3\ kmole^{-1}\ d^{-1}$	10^{10}	Batstone et al. (2002)[7]
$K_{A/B,ac}$	S_{ac} acid-base kinetic parameter	$m^3\ kmole^{-1}\ d^{-1}$	10^{10}	Batstone et al. (2002)[7]
$K_{A/B,CO_2}$	S_{CO_2} acid-base kinetic parameter	$m^3\ kmole^{-1}\ d^{-1}$	10^{10}	Batstone et al. (2002)[7]
$K_{A/B,IN}$	S_{IN} acid-base kinetic parameter	$m^3\ kmole^{-1}\ d^{-1}$	10^{10}	Batstone et al. (2002)[7]
$K_{a,bu}$	S_{bu} acid-base equilibrium parameter	$kmole\ m-3$	$1.51\ 10^{-5}$	Batstone et al. (2002)[7]
$K_{a,ac}$	S_{ac} acid-base equilibrium parameter	$kmole\ m-3$	$1.74 \cdot 10^{-5}$	Batstone et al. (2002)[7]
K_{a,CO_2}	S_{CO_2} acid-base equilibrium parameter	$kmole\ m-3$	$4.94 \cdot 10^{-7}$	Batstone et al. (2002)[7]
$K_{a,IN}$	S_{IN} acid-base equilibrium parameter	$kmole\ m-3$	$1.11 \cdot 10^{-9}$	Batstone et al. (2002)[7]
K_{H,H_2}	Henry's law coefficient of S_{H_2}	$kmole\ m^{-3}\ bar^{-1}$	$2.72 \cdot 10^{-2}$	Batstone et al. (2002)[7]
K_{H,CO_2}	Henry's law coefficient of S_{CO_2}	$kmole\ m^{-3}\ bar^{-1}$	$7.3847 \cdot 10^{-4}$	Batstone et al. (2002)[7]
kLa	gas-liquid transfer coefficient	d^{-1}	200	Batstone et al. (2002)[7]
R	Gas law constant	$bar\ m^3\ kmole^{-1}\ K^{-1}$	0.083145	Batstone et al. (2002)[7]
T	Temperature within the reactor	K	308	Experimental value
pH_{UL}	pH upper limit	$--$	5.5	Batstone et al. (2002)[7]
pH_{LL}	pH lower limit	$--$	4	Batstone et al. (2002)[7]
K_{IN}	S_{IN} affinity constant for X_{su}	$kgCOD\ m^{-3}$	$1 \cdot 10^{-4}$	Batstone et al. (2002)[7]
K_L	Leaching inhibition constant	$kg\ m^{-3}$	5.6	Calibrated value
$Time$	Simulation time	d	16	Experimental value

Table S2. Kinetic, stoichiometric, and operating parameters.

References

1. Association, A. P. H., Association, A. W. W., Federation, W. P. C. & Federation, W. E. *Standard methods for the examination of water and wastewater*, vol. 2 (American Public Health Association., 1912).

2. Ghimire, A. et al. A review on dark fermentative biohydrogen production from organic biomass: process parameters and use of by-products. *Appl. Energy* **144**, 73–95 (2015).

3. Yao, Y.et al. Hydrometallurgical processes for recycling spent lithium-ion batteries: a critical review. *ACS Sustain. Chem. & Eng.* **6**, 13611–13627 (2018).

4. Le, M. N. & Lee, M. S. A review on hydrometallurgical processes for the recovery of valuable metals from spent catalysts and life cycle analysis perspective. *Miner. Process. Extr. Metall. Rev.* 1–20 (2020).

5. Ghassa, S., Farzanegan, A., Gharabaghi, M. & Abdollahi, H. The reductive leaching of waste lithium ion batteries in presence of iron ions: Process optimization and kinetics modelling. *J. Clean. Prod.* **262**, 121312 (2020).

6. Russo, F. et al. Sustainable asphalt mastics made up recycling waste as filler. J. Environ. Manag. 301, 113826 (2022).

7. Batstone, D. J.et al. The iwa anaerobic digestion model no 1 (adm1). *Water Sci. technology* **45**, 65–73 (2002).
